# Predictive Values of the CatLet© Angiographic Scoring System for 30-Day Cardiac Mortality in Patients after Primary Percutaneous Coronary Intervention

**DOI:** 10.31083/RCM28198

**Published:** 2025-03-17

**Authors:** Chenjie Zhang, Wenhui Liang, Zongliang Yu, Yongming He

**Affiliations:** ^1^Department of Cardiology, The First People’s Hospital of Kunshan, 215300 Kunshan, Jiangsu, China; ^2^Department of Cardiology, The First Affiliated Hospital of Soochow University, 215000 Suzhou, Jiangsu, China

**Keywords:** CatLet© score, cardiac mortality, acute myocardial infarction, short-term prognosis

## Abstract

**Background:**

The Coronary Artery Tree Description and Lesion Evaluation (CatLet©) angiographic scoring system is a newly developed tool to predict the long-term clinical outcomes for patients with acute myocardial infarction (AMI). This study aimed to evaluate the predictive value of this novel angiographic scoring system for cardiac mortality in AMI patients within 30 days of primary percutaneous coronary intervention (pPCI) in AMI patients.

**Methods:**

Patients with AMI undergoing pPCI were consecutively enrolled between January 2012 and July 2013. The CatLet© score was calculated for all the lesions in the non-occlusive status and were tertile partitioned into three groups: CatLet-low ≤14 (N = 124), CatLet-mid 14–22 (N = 82), and CatLet-top ≥22 (N = 102). The primary endpoint was cardiac mortality at 30 days after the procedure. Survival curves were generated using the Kaplan-Meier method, and survival rates among the CatLet© score tertiles were compared using the Log-rank test. Furthermore, Cox regression analysis was performed to identify the associations between the predictors and clinical outcomes.

**Results:**

A total of 308 patients were included in the final analysis. The included patients were followed up for 30 days, with 19 (6.17%) cardiac death. Kaplan-Meier curves indicated that the CatLet-top tertile exhibited a significant increase in the risk of cardiac mortality when compared with the low and mid tertiles (*p* for trend <0.01); the CatLet© score remained an independent predictor of 30-day cardiac mortality in AMI patients after adjusting for clinical variables (HR (95% CI): 6.13 (1.29–29.17); *p* < 0.01). The multivariable analysis demonstrated that a per 1 unit increase in CatLet© score was associated with a 1.04 (1.01–1.06)-fold increased risk of cardiac death. The area under the receiver operating characteristic (ROC) curve (AUC) statistic for the CatLet© score was 0.80 (95% CI, 0.69–0.91), with a good calibration (χ^2^ = 12.92; *p* = 0.12).

**Conclusion:**

The CatLet© score can be used to predict the short-term cardiac death in AMI patients. A CatLet© score ≥22 or ≥11 myocardial segments involved relative to the total 17 segments (the score divided by 2), including culprit or non-culprit vessels, accounting for 65% (11/17) of left ventricle mass involved, is significantly associated with poor prognosis. The current study has extended the application of the CatLet© score in clinical practice.

**Clinical Trial Registration:**

ChiCTR-POC-17013536. Registered 25 November, 2017, https://www.chictr.org.cn/showproj.html?proj=22814.

## 1. Introduction

Risk scores derived according to clinical variables, anatomic variables or both have been widely used for diagnosis, risk-stratification, decision-making, and outcome prediction for patients with coronary artery disease [1, 2, 3, 4]. The SYNergy between percutaneous coronary intervention (PCI) with TAXUS™ and Cardiac Surgery (SYNTAX) score and Gensini scores, based on the anatomic variables, have been widely used and validated to be able to risk-stratify and make the plan on the revascularization strategy [5, 6]. However, none of these anatomic prediction models has taken into account the variability in coronary anatomy among individuals. The variability of the left anterior descending artery (LAD), diagonal, or right coronary arteries is at random in individuals, the continuity of this variability, however, can be observed in a whole population, which offers the possibility of quantifying or semi-quantifying this variability. For example, the right coronary artery (RCA) can be so small that it does not supply blood to the left ventricle at all or can be large enough to supply blood to the entire inferior wall or even the obtuse margin of the left ventricle. Our research team has recently developed the Coronary Artery Tree Description and Lesion Evaluation (CatLet©) score based on the 17-segment myocardial model, the law of competitive blood supplies, and the law of flow conservation. This scoring system is unique in that it can be used to interpret the variability in coronary arteries, assess the severity of lesion stenosis, and estimate the extent of myocardial territory involved [7]. Previous studies have shown that the CatLet© score predicts long-term clinical prognosis with a high reproducibility [8, 9, 10, 11]. However, its predictive value for short-term clinical prognosis has not been elucidated.

## 2. Methods

### 2.1 Study Subjects

**Fig. 1.**
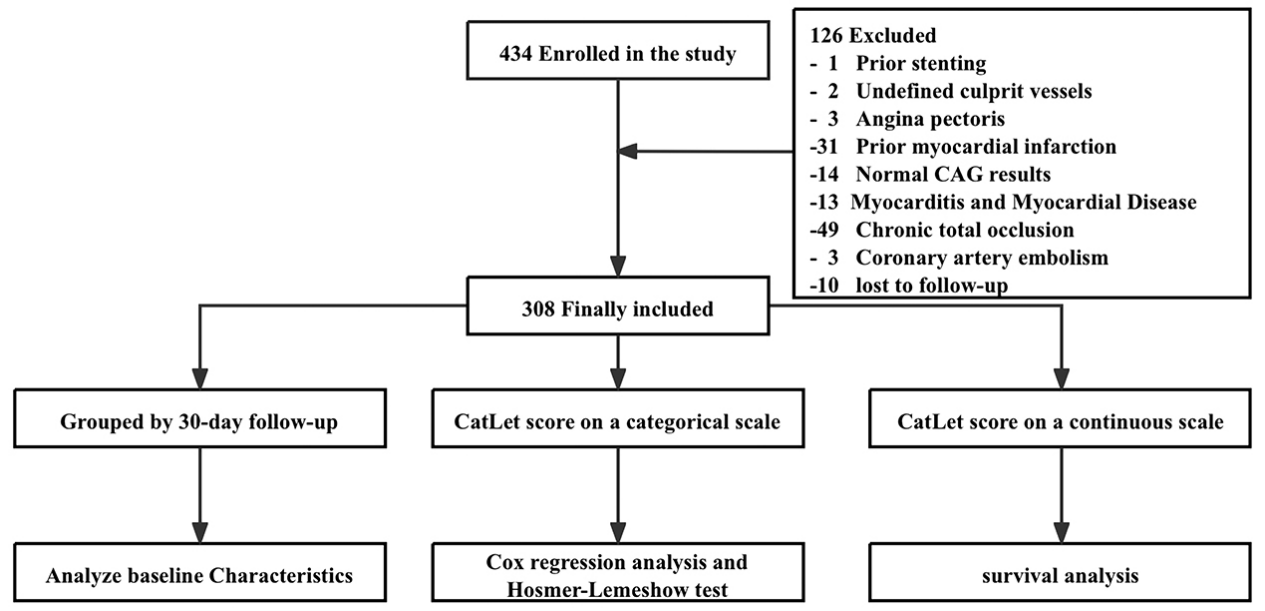
**Flowchart of the study**. Abbreviations: CAG, coronary angiography.

A total of 434 patients who visited the First Affiliated Hospital of Soochow University with primary percutaneous coronary intervention (pPCI) owing to chest pain ≤12 hours after symptom onset, from January 2012 through July 2013, were consecutively included in this study. 126 patients were excluded, and the exclusion criteria were detailed in Fig. 1. A total of 308 patients were finally included for analysis.

### 2.2 Clinical Data

The electronic medical record system was retrieved to obtain the following information: (1) demographic characteristics: name, gender, age; (2) medical history: hypertension, type 2 diabetes, history of stroke; (3) smoking history and alcohol consumption history; (4) biochemical tests: blood creatinine, ejection fraction, lipoprotein(a); (5) cardiac function tests: echocardiography as well as standard 12-lead electrocardiogram; (6) coronary angiography records: number of lesions, “culprit” vessels, treated vessels, coronary circulation pattern, Medina types [12], and adverse angiographic characteristics pertaining to the lesions.

### 2.3 CatLet© Angiographic Scoring System

#### 2.3.1 Coronary Reclassification

Previous study has detailed the nomenclature and reclassification of coronary arteries in the CatLet© score [4]. RCA is reclassified into six types from smallest to largest: posterior descending artery (PDA) zero, PDA only, small RCA, average RCA, large RCA, and super RCA; LAD, into three types: short LAD, average LAD, and long LAD; and diagonal branches (Dx), into three types: small Dx, inter. Dx, and large Dx, which together results in a total of 54 (6 × 3 × 3) types of coronary circulation pattern to describe coronary artery variability. Each vessel segment under a particular coronary circulation pattern is assigned a corresponding weighting factor. The presence of ≥50% diameter stenosis in coronary vessels ≥1.5 mm in diameter was defined as a coronary lesion, with a multiplicative factor of 2.0 for lesions with 50–99% luminal reduction. Downstream vessels that were persistently invisible after wiring or small ballooning were regarded as entirely occluded lesions, with a multiplicative factor of 5.0. The CatLet© score can be accessible at http://www.catletscore.com/ [5].

#### 2.3.2 Illustration of the Scoring Process

**Fig. 2.**
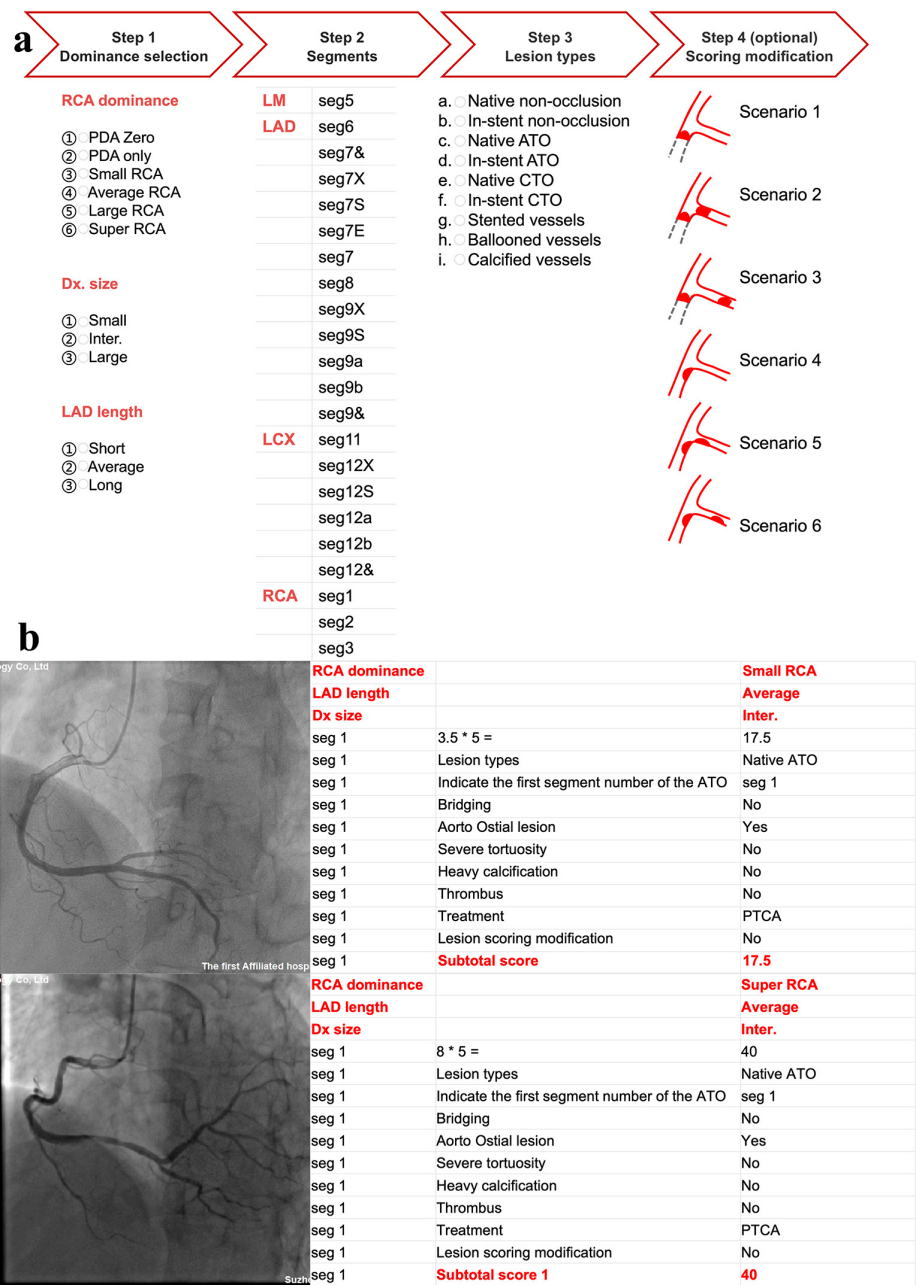
**Illustration of the scoring process in two representative cases**. (a) The scoring process. The CatLet© score is obtained by completing the four steps prompted by the webpage. (b) Illustrates a typical example of the Coronary Artery Tree Description and Lesion Evaluation (CatLet©) score. The angiographic images show a small RCA and a super RCA, all with inter. Dx and average LAD. Assume that the proximal RCA, i.e., seg 1, is completely occluded, the CatLet© score for small RCA and super RCA are 17.5 and 40 points, respectively. In the traditional dominance classification, these two RCA types are taken as the same. Abbreviations: PDA, posterior descending artery; RCA, right coronary artery; Dx, diagonal branches, ATO, acute total occlusion; CTO, chronic total occlusion; PTCA, percutaneous transluminal coronary angioplasty; LAD, left anterior descending artery; LM, left main; LCX, left circumflex artery.

Using the calculator on the webpage of http://www.catletscore.com/, the score can be obtained in 4 steps (Fig. 2): Step 1, select the type of RCA, Dx and LAD to derive the coronary circulation pattern; Step 2, select the affected segments of the diseased vessels for a lesion; Step 3, select the type of stenosis of the diseased vessels; and Step 4, in the case of a mother-daughter relationship with respect to blood supply, the score correction needs to be considered. Those adverse angiographic characteristics were collected but not scored, such as Bifurcation lesions, trifurcation lesions, lesions length >20 mm, distortions, etc.

### 2.4 Primary Endpoint and its Definition

The primary endpoint was cardiac death. At 30 days of follow-up, the cause of death for all patients was cardiac, without other major adverse cardiovascular events, such as stroke, non-fatal myocardial infarction, unplanned revascularization occurring during this period. Cardiac deaths were defined using the definitions recommended by the Academic Research Consortium: (1) deaths due to myocardial infarction, heart failure, and fatal arrhythmias; (2) unwitnessed deaths and unexplained deaths; and (3) all deaths related to percutaneous coronary interventions or coronary artery bypass grafting procedures [13].

### 2.5 Sample Size Estimation and Statistical Analysis

PASS 15.0.5 (NCSS LLC, Kaysville, UT, USA) was used to estimate the sample size required for this study. According to previously reported 30-day post-PCI mortality rates of 4.5–10% in acute myocardial infarction (AMI) patients [14, 15], the mean value of 7% was used to estimate the sample size for this study. Our previous study showed a 6% increase in the risk of cardiac death for each 1-unit increase in CatLet© score [8]. With a power of 0.8 and a two-sided α = 0.05; therefore, at least 268 AMI patients are required to draw a reliable conclusion. In this study, a total of 308 AMI patients were finally included. Data analysis and plotting were completed using STATA 15.0 (State Corp LP, College Station, TX, USA). Missing values were handled by multiple imputation with 25 times. Continuous variables were tested for their normality by the Shapiro-Wilk test: Continuous variables that were not normally distributed were expressed as Median (interquartile range, IQR), and comparisons between groups were made using the Wilcoxon rank-sum test; Continuous variables that were normally distributed were expressed as mean ± standard deviation, and comparisons between groups were made using the independent samples *t*-test. Categorical variables were expressed as counts and frequencies (%), and comparisons between groups were made using the chi-squared test. Kaplan-Meier survival curves were plotted, and Log-rank tests were used to compare survival rates. Cox regression models were constructed for multivariable analysis of the effect of the CatLet© score on short-term clinical prognosis, and the area under the receiver operating characteristic (ROC) curve and the Hosmer-Lemeshow test were used to evaluate the discriminatory and calibration of the models. All tests were two-sided, and a *p* value of <0.05 was considered statistically significant.

## 3. Results

### 3.1 Baseline Characteristics

At 30 days of follow-up, there were 19 deaths, accounting for 6.17% of the total. Compared with the non-event group, the cardiac death group was older, had higher CatLet© score, higher creatinine levels, more never-smokers, and a higher mortality rate in women (*p* < 0.05), shown in Table 1.

**Table 1.** **Baseline characteristics**.

		Missing	Non-event	Cardiac death	*p* value
N			289	19	
Male			54 (18.7%)	9 (47.4%)	<0.01
Age, years			63.00 (17.00)	80.00 (15.00)	<0.01
CatLet© score			16.50 (12.00)	32.00 (26.00)	<0.01
Hypertension			161 (55.71%)	14 (73.68%)	0.13
Diabetes			63 (21.80%)	7 (36.84%)	0.13
Cr, mold/L		4 (1.3%)	7.20 (2.40)	11.92 (7.03)	<0.01
Lap(a), mg/L		16 (5.19%)	99.50 (135)	99.50 (353.00)	0.30
LVEF		6 (1.95%)	48.52 ± 0.53	45.74 ± 2.32	0.10
STEMI			270 (93.43%)	18 (94.74%)	0.82
Smoking		21 (6.82%)			<0.01
	Never		93 (32.18%)	14 (73.68%)	
	Past		21 (7.27%)	0 (0.00%)	
	Current		175 (60.55%)	5 (26.32%)	
Alcohol consumption		21 (6.82%)			0.18
	Never		206 (71.28%)	18 (94.74%)	
	Past		10 (3.46%)	0 (0.00%)	
	Current		73 (25.26%)	1 (5.26%)	
No. of lesion/patient			2.06 ± 1.15	2.95 ± 1.75	0.02
Coronary artery dominance					
RCA size					0.04
	PDA zero		19 (6.6%)	2 (10.5%)	
	PDA only		17 (5.9%)	4 (21.1%)	
	Small RCA		76 (26.3%)	4 (21.1%)	
	Average RCA		99 (34.3%)	2 (10.5%)	
	Large RCA		67 (23.2%)	5 (26.3%)	
	Super RCA		11 (3.8%)	2 (10.5%)	
Diagonal size					0.93
	Small		44 (15.2%)	3 (15.8%)	
	Inter.		189 (65.4%)	13 (68.4%)	
	Large		56 (19.4%)	3 (15.8%)	
LAD length					0.78
	Short		34 (11.8%)	3 (15.8%)	
	Average		194 (67.1%)	13 (68.4%)	
	Long		61 (21.1%)	3 (15.8%)	

Abbreviations: LVEF, left ventricular ejection fraction; STEMI, ST-elevated myocardial infarction; Cr, creatinine; Lp(a), lipoprotein (a).

### 3.2 Distribution of CatLet© Score

**Fig. 3.**
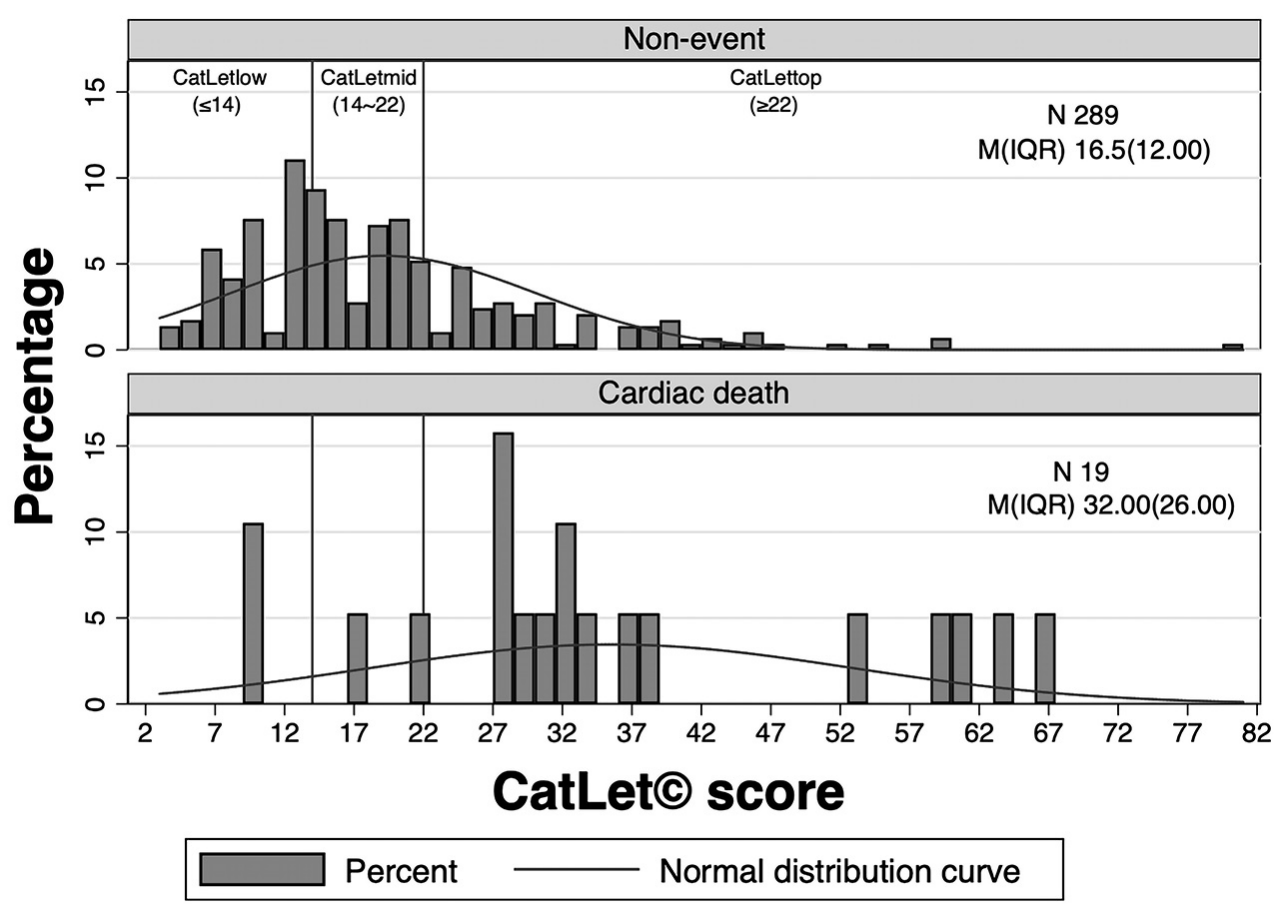
**The distribution of CatLet© score**. Abbreviations: IQR, interquartile range; M, median.

A total 308 patients had a mean CatLet© score of 20.11 ± 12.06. The median scores were 16.50 and 32.00 in the non-event group and in the cardiac-death group, respectively. Patients in the non-event group had CatLet© score primarily clustered in the low and mid CatLet© score. In contrast, patients in the cardiac-death group mainly had been distributed in the top CatLet© score (*p* < 0.01) as shown in Fig. 3.

### 3.3 Short-Term Prognosis

**Fig. 4.**
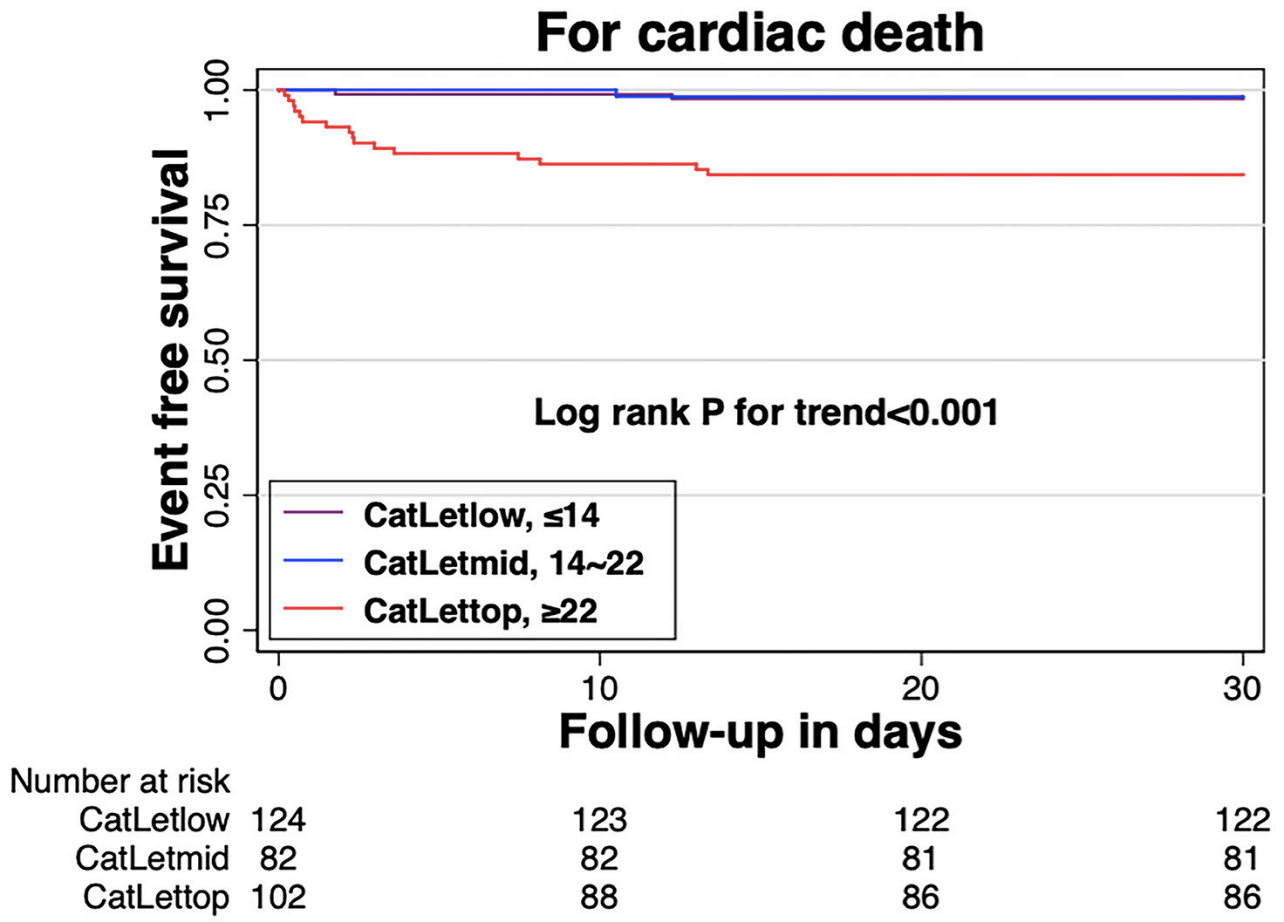
**Kaplan-Meier curves for cardiac death according to CatLet© score tertiles**.

**Fig. 5.**
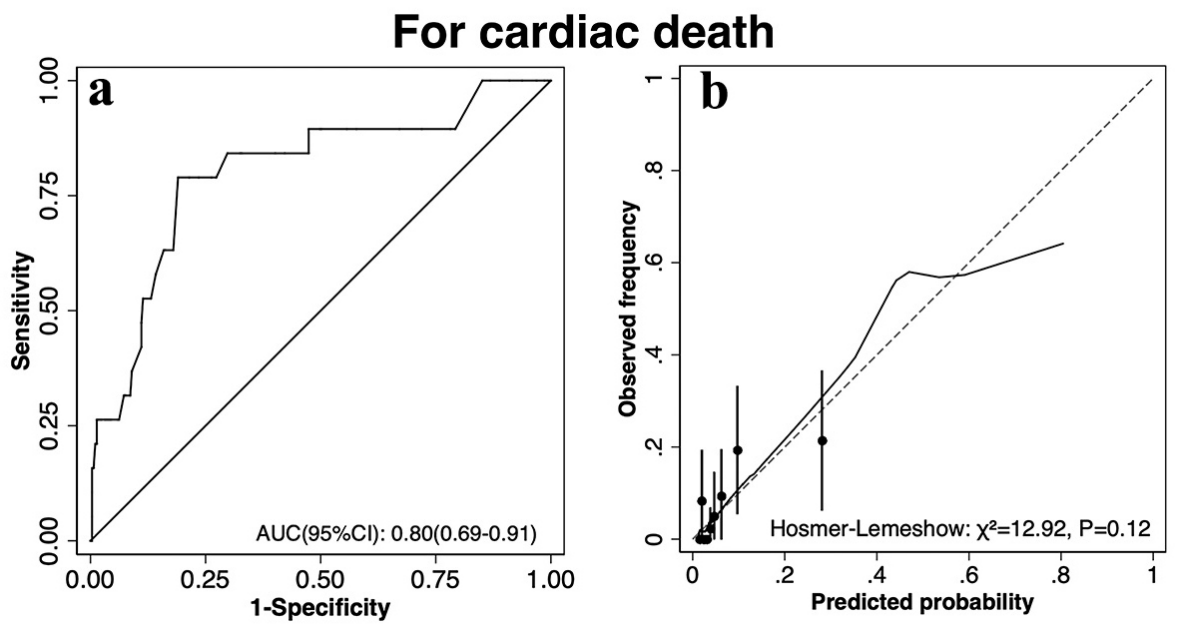
**The discrimination (a) and calibration ability (b) of CatLet© score for prediction of cardiac death**. Abbreviations: AUC, area under the receiver operating characteristic (ROC) curve.

The CatLet© score was tertile partitioned: the CatLetlow ≤14 (N = 124), the CatLetmid 14–22 (N = 82), and the CatLettop ≥22 (N = 102). The 30-day mortality was 1.61% and 1.22% in the low and mid groups, respectively, whereas it was as high as 15.69% in the top group. The Kaplan-Meier survival curves (Fig. 4) showed a significant decrease in survival in the top group compared to the low/mid group (*p* for trend <0.01). Table 2 showed that patients in the top group had a 10.53-fold higher risk of cardiac death compared to the low group (95% CI: 2.42–45.82, *p* < 0.01). After controlling for risk factors, patients in the top group had a 6.13-fold increased risk of cardiac death compared with the low group (95% CI: 1.29–29.17, *p* < 0.01). The multivariable-adjusted risk for cardiac death per 1-unit increase in CatLet© score was 1.04 (1.01–1.06), as detailed in **Supplementary Fig. 1**. The area under the ROC curve (AUC) for the CatLet© score was 0.80 (0.69–0.91), A CatLet© score of 22 corresponds to a sensitivity of 78.95% (54.4–93.9%) and a specificity of 72.66% (67.1–77.7%) for prediction of cardiac death; the model was well-calibrated (χ^2^ = 12.92, *p* = 0.12), as detailed in Fig. 5.

**Table 2.** **Univariate and multivariable-adjusted HRs for cardiac death on a categorical and continuous scale CatLet© score**.

	Cardiac death	HR (95% CI)	*HR (95% CI)
CatLet© score on a categorical scale			
CatLetlow	2 (1.61%)	1.0	1.0
CatLetmid	1 (1.22%)	0.75 (0.07–8.31)	0.60 (0.05–7.54)
CatLettop	16 (15.69%)	10.53 (2.42–45.82)	6.13 (1.29–29.17)
*p* for trend	<0.01	<0.01	<0.01
CatLet© score on a continuous scale			
CatLet© score		1.06 (1.04–1.08)	1.04 (1.01–1.06)

*HR: Adjusting for age, sex, alcohol consumption, smoking, hypertension, blood creatinine, left ventricular ejection fraction, stroke and diabetes mellitus.

## 4. Discussion

In this study, we have found that AMI patients with CatLet© score ≥22 or more than 11 segments involved relative to the 17 segments in total (the score divided by 2) has a significantly higher risk of cardiac death within 30 days. The CatLet© score is an independent predictor of short-term clinical prognosis in patients with AMI undergoing pPCI.

Previous studies have shown that the 30-day mortality rate in patients with AMI treated with pPCI is 4.5–10% [1, 14, 15, 16, 17]. The present study had a similar mortality of 6.17%. We found that both the PDA only and the super RCA types were more common in the cardiac death group. In contrast, the average RCA was more common in the non-event group. The possible explanation is that when the coronary arteries are evenly distributed, the myocardium at jeopardy by occlusion of one of the coronary arteries is relatively limited; when the coronary artery distribution is extremely distributed, occlusion of the dominant vessel results in considerable myocardial ischemia or necrosis, which makes the recovery of cardiac function postoperatively challenging and leads to a poorer prognosis for patients.

The CatLet© score of 22 or 11 myocardial segments involved relative to the 17 segments in total is the cutoff for the top tertile, accounting for 65% of the total left ventricle. Clinically meaningful cutoff points still require further study in large sample size populations. According to the CatLet© score, the lesion score is the product of the vascular weighting factor (extent of blood supply) and the stenosis multiplication factor. Previous studies have shown that both the degree of stenosis and myocardial infarcted territory are associated with clinical prognosis [18, 19]. Therefore, it is not surprising that the CatLet© score, which takes into account both the degree of stenosis of the lesion and the extent of its blood supply, can predict the prognosis of patients with AMI. The SYNTAX score, a widely used score of the coronary arteries, has a C-index ranging 0.60–0.78 in prediction of short-term cardiac death in patients with AMI [20, 21, 22]; the AUC value of the CatLet© score in the present study was 0.80 (0.69–0.91), which is superior to the SYNTAX score. Previous study has similarly shown [8] that the CatLet© score is superior to SYNTAX in predicting the long-term prognosis of patients with AMI. The excellent performance of the CatLet© score with respect to outcome predictions for patients with AMI may be that this novel angiographic scoring system has taken into account the variability in coronary anatomy and more accurately identified the myocardial territory at jeopardy as compared with the SYNTAX score.

The present study has some limitations. Firstly, this study only considered the relationship between the extent of vascular lesions and short-term prognosis and did not include clinical factors, and our previous study have shown that the addition of clinical factors contributes to model improvement [9], and the present study also found that after adjusting for covariates, age was still an independent predictor of short-term cardiogenic death. Therefore, both clinical and angiographic variables have adversely affected the clinical outcomes, which should be considered in clinical practice; secondly, although the basis of sample size estimation was given in the present study, it is indisputable that the sample size was moderate. Therefore, a large sample size population is still needed to confirm the value of CatLet© score in the short-term prognosis of patients with AMI; finally, this study failed to document the door-balloon time, a baseline information related to prognosis. But, the present study only enrolled patients with AMI and undergoing pPCI, with chest pain within 12 h since symptom onset, which minimized the effects of this confounding factor on our main findings.

## 5. Conclusion

CatLet© score has a predictive value for short-term clinical prognosis in patients with AMI, and the risk of cardiac death is significantly high in AMI patients with CatLet© score ≥22 points or more than 11 myocardial segments involved, which expands the clinical application of this score in clinical practice. Higher CatLet© score or more segments involved relative to the 17 segments in total have meant more aggressive treatment strategy possibly needed. A large sample size study is warranted to validate this finding in the current study.

## Availability of Data and Materials

The datasets used and analyzed during the current study are available from the corresponding author on reasonable request.

## Supplementary Material

Supplementary material associated with this article can be found, in the online version, at https://doi.org/10.31083/RCM28198.
