# Supplementary material for: Predictive Values of the CatLet© Angiographic Scoring System for 30-Day Cardiac Mortality in Patients after Primary Percutaneous Coronary Intervention
Source: Rev Cardiovasc Med. 2025 Mar 17;26(3):28198. doi: 10.31083/RCM28198 (PMC11951280; doi:10.31083/RCM28198)
Supplement: Supplementary file 1 [file 2153-8174-26-3-28198-s1.docx]

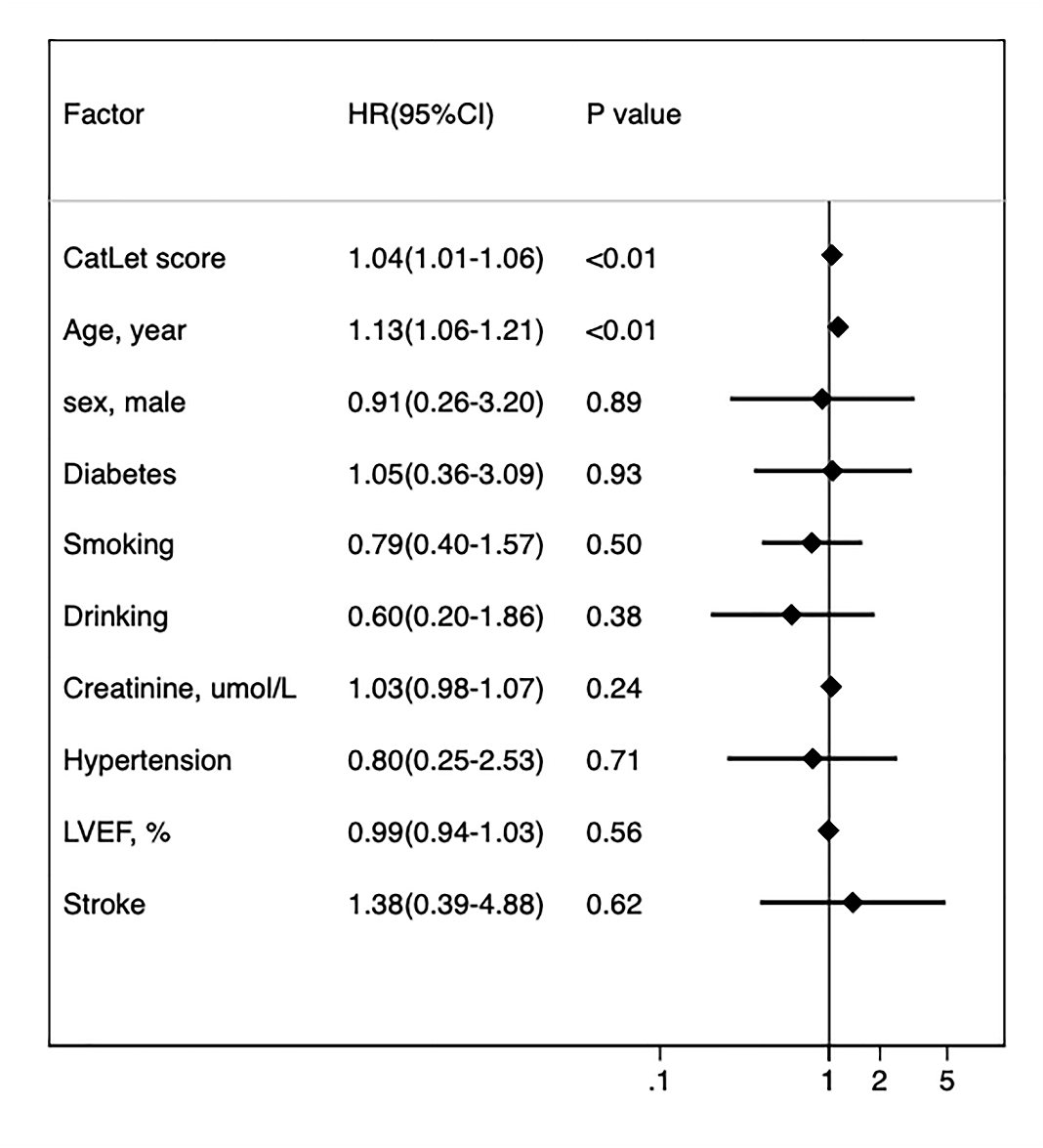


Fig.S1 Multivariable predictor of 30-day cardiac death

HR: Hazard Ratio, CI: Confidence Interval, LVEF: left ventricular ejection fraction
